# Supplementary material for: Identification of neural progenitor cells and their progeny reveals long distance migration in the developing octopus brain
Source: eLife. 2021 Aug 24;10:e69161. doi: 10.7554/eLife.69161 (PMC8384421; doi:10.7554/eLife.69161)
Supplement: Supplementary file 1. — N/A, not applicable; TR, technical replicate. [file elife-69161-supp1.docx]

# Supplementary file 1

**Table S1. Number of biological replicates (unless specified otherwise) for immunohistochemistry and *in situ* hybridization experiments.** *Abbreviations: N/A, not applicable; TR, technical replicate*

|  | **PH3, AcTub** | ***Ov-soxB1*** | ***Ov-ngn*** | ***Ov-ascl1*** | ***Ov-neuroD*** | ***Ov-elav*** | ***Ov-syt*** | ***Ov-pnca*** |
| --- | --- | --- | --- | --- | --- | --- | --- | --- |
| **Stage VII.2** | 2 | N/A | N/A | N/A | N/A | N/A | N/A | 2 |
| **Stage IX** | 2 | 1 | 2 (TR)  + 2 (TR) | 1 | 1 | 2 (TR)  + 2 | 2 | 2 |
| **Stage XI** | 2 | 4 | 4 | 1 | 1 | 4 | 3 | 4 |
| **Stage XIII** | 2 | 4 | 2 (TR)  + 4 | 2 | 2 | 2 | 2 | 2 |
| **Stage XV.2** | 2 | 2 | 2 (TR)  + 2 | 1 | 2 | 1 | 1 | 2 |
| **Stage XVII** | 1 | 3 | 1 | 3 | 3 | 3 | 1 | 2 |
| **Stage XIX.1** | 1 | 2 | 2 | 3 | 3 | 2 | 1 | 1 |
| **Stage XX.2** | 3 | 2 | 1 | 1 | 1 | 2 | 3 | 1 |
