# Supplementary material for: Identification of neural progenitor cells and their progeny reveals long distance migration in the developing octopus brain
Source: eLife. 2021 Aug 24;10:e69161. doi: 10.7554/eLife.69161 (PMC8384421; doi:10.7554/eLife.69161)
Supplement: Supplementary file 2. [file elife-69161-supp2.docx]

# Supplementary file 2

**Table S2. Nucleotide sequence of primers used to amplify gene fragments for ISH probes**

|  | **Forward (5' -> 3')** | **Reverse (5' -> 3')** |
| --- | --- | --- |
| ***Ov-ascl1*** | AAGCAGCGGCACGAAATA | CGGTGTCACTATCCTCAACAAG |
| ***Ov-elav*** | GAACACACTGAGACAGAATGA | CCAAAGAGACGCCAAAGT |
| ***Ov-neuroD*** | TGGTGACAGCAGTAGCGAGT | TGAAGACATCCAGCAACGAG |
| ***Ov-ngn*** | TCATCTTCGTCGCTGTCATTT | CTCAGGGTCCAGATGTAATTG |
| ***Ov-pcna*** | ATGGGACTGTAGTTCAACAGGAA | GGTGCCAGATAATATCGGATGTA |
| ***Ov-soxB1*** | CAGTCGCAGAAGAACAACCA | ATTTGAGGCACCTGAGATGG |
| ***Ov-syt*** | GCAGGAGAGGGAAGAAAGATG | CTCCAATGACGCAATCCAGTA |
