# Supplementary material for: Identification of neural progenitor cells and their progeny reveals long distance migration in the developing octopus brain
Source: eLife. 2021 Aug 24;10:e69161. doi: 10.7554/eLife.69161 (PMC8384421; doi:10.7554/eLife.69161)
Supplement: Supplementary file 3. [file elife-69161-supp3.docx]

# Supplementary file 3

**Table S3. Nucleotide sequence of probes for colorimetric *in situ* hybridization**

| *Ov-ascl1* | AAGCAGCGGCACGAAATACAATGACAGCGGCACTGGTGCCGGAACCAATGGTGGTAGAGTCGACGCTGACGTCATCGGCTACAACCACGACAACGACGGCGACTGCGATGATGGCTGCGACGACGACGACGAACGCGTTTTCAACGACGGCGAAAGCATTTTCAACGACCACGGCAGTGGCGGCAACGAACGGGTTTCCAACGACGACGACGACGGCGGCGACCACAAACTCCACAACGACGACGACGACTCCCAATTCGAAATCAGTCCGTACGATATCGATGAAAGAGAACGCACGCGAGCTGATGAACTGTAAGCGACGTCTTGATTTCAACCACACCAACTACTTGCCGTTGCATCGACCACAGACAGTTGCCGTGGCTCGGCGCAACGAACGCGAAAGGAACCGCGTTAAGTTGATCAACATGACTTTTGCCACGCTTAGAGAACATATACCGAACGGTCCGAAGGGCAGTAAGAACAAAAAAATGAGCAAAGTAGAAACTTTACGAGCAGCCATTGACTACATCCACTATCTACAGGACCTTGTTGAGGATAGTGACACCG |
| --- | --- |
| *Ov-elav* | GAACACACTGAGACAGAATGATTTAGATCAAGGACTGCCGGAAACAGCTAACACTGACTTTCACTGTGACCTGCTTCCGGCAGCACGAAATGCATCAACCGATGCGGAAAAATGTCAGAGTTTAGGTTATGGCTTTGTTAACTACAAATACCCTAGTGATGCAGAAAAGGCAATCAATACTTTAAATGGATTGAGATTACAAAATAAAACAATAAAGGTTTCATATGCCCGACCAAGCAGTGAAAGTATTAAAGGGGCAAACTTGTACATAAGTGGCTTGCCAAAGTCTTTCACTCAGTTGGACTTGGAGAAATTGTTTTCTCAGTGTGGATTCATTATAACATCCAGAATTCTTTATGACAATAACACAGGCTTATCCAAAGGTGTTGGCTTCATCCGATTTGATCAACGAATCGAAGCTGAAAGAGCTATCCAAAAATTACACGGTACCATTCCTGAAGGAGCAACTGAACCTATTACTGTAAAGTTTGCTAACTCTCCAAGCTCAAATAAAAATGCTGTACCTCTAGCTTTAGCAGCTTCATATTTATCACCAAGTAGGAGAATCTTTGGTCCAATTCATCATGCTGCTGGAAGATTCAGATACTCACCATTGGAAGCTAGTCTACTGCCAGGAACAATACTTCCAGGGAATGCTCTAAATGGCACTGGTTGGTGTATTTTTGTATACAACCTTGCACCAGACACTGAGGATGATGTACTTTGGCGTCTCTTTGG |
| *Ov-neuroD* | TGGTGACAGCAGTAGCGAGTTGTTGGGTGATGGTTCTTTGCTAGATGACACATGTTCAGAAGATGACTTGGATGATTATGGCAGCGAACCAGAAGATTGCCTGGAAGGCATGACTGGTAATGAAGACGAACAAGAAATGAGTGATACAAAGATGAAGAAAAGCGCGAAAAACTATCCTTACCGCAGGAAGAAGGAAGAGAAACCGTCTTCCAAAGACAAGAACGAACCACGTCTTCCAAAGAAACGTGGTCCAAAGAAGAAACGGATGACAAAAGCGCGAGTGCAAAAATTACGCTTGCGACGAGTTAAAGCAAATGCACGAGAACGCAACCGCATGCATGGTTTGAACGATGCATTGGACACTTTGCGATTGCACGTGCCATGCACGTCCAAGACACAGAAGCTATCGAAAATTGAAACGTTACGATTGGCACGCAATTATATCAGTGCCTTGGGAGAAATTTTAAAAAATGGTACTAAACCAGATGGTGTCAGTTTTGCGAAAGCTTTGTCAAAAGGTCTTTCTCAAAACACAATGAATCTCGTTGCTGGATGTCTTCA |
| *Ov-ngn* | TCATCTTCGTCGCTGTCATTTTCTCCTCTTTATACCGGCGACCACAAAGTGCCCGCGTTAAAATCGAACCTGCAACAGTTATTACCACAAAACCAACCGCAAAATGAACGACAGCCTGAGTCTCAGCTTCAGCAAGAGTCGAAACCGCAACCTCAGCAACAACCGGATATGAAGGCAGCCGTTGCATGTCCGGTTTTGTTACCCACCTCCGCGGCACAGTGCAAGGATACTGTCGTGCCAACGAACTCTATTAACACAACTGCAGAAACAACAACAACAACAGCATCATCATCATCATCATCATCATTAGCAGCACCAAGAACAAAAGCAACAACAACAAGGAGAAAAATAACGGAAATGACAAAAGCAACGAGACCACCAATAATATCACGATCACCACCAACATCATCATCATCATCATCATCATCATCATCATTATCGTCCTCTTCACCGTTAACGTCTCGTGCATCTCCATTATCATCATCATCACCACCATCGTCAATACCAATAATATCCTCGTCAACTGATTCCGTTGACGGGTTGAAAAGAAAACAAGTAAGTCCGGAAAAAAACTGCGGCGCGAAGCGACGTAACGGACCCACGGCAAAGAAAAAGCGTTACACCAAGTCACGGATTCGAAACTGTAACCCTTTGATTGTGATGAAAATCAAGAGAAACAGACGAATGAAAGCCAACGACCGAGAACGTTCTCGAATGCACAGCTTAAACGATGCTTTGGATTGTCTTCGAGAAGTTCTACCAAATTATTCAGACGAAGGGAAATTGACCAAAATTGAAACGCTGCGTTTCGCCCACAATTACATCTGGACCCTGAG |
| *Ov-pcna* | ATGGGACTGTAGTTCAACAGGAATAAGTTTACAAGCTATGGATTCATCACATGTTAGTTTAGTTTCTTTATTATTACGTGCAGATGGCTTTGATACTTACAGATGTGATCGCAACTTGTCAATGGGCATCAACCTCAACAGCATGTCCAAGATCCTCAAATGTGCCTGCAATGATGATATAATCACAGTGAAAGCTGAAGATAATGCTGATACAGTCACTTTCGTTTTTGAATCTCCAAATCAAGAAAAAGTCTCTGATTATGAAATTAAATTGATGGACATTGATTCTGAACATTTAGGTATTCCTGATACTGAGTACAACTGTGTTGTAAAACTTCCTTCAGCTGAATTCCAAAGAATATGTCGAGATTTGAGCCAAATTGGTGATTCTGTTGTGATTTGTTGTACAAAAGAAGGAGTTAAATTCTCAGCCAGTGGTGATCTTGGATGTGGTAACATCAAGTTAGCCCAAAGTACCAATGCTGATAAAGAGGAAGAGTCTGTCACTATTGAAATGAACCAGTCTGTTGTGCTGACATTTGCTCTGCGATATTTGAATTTCTTCACCAAAGCTACACCATTGTCTACTCATGTTACACTCTCTATGTCACAAGACGTTCCACTTGTGGTGGAATACAAAATAGCTGATACAGGTTACATCCGATATTATCTGGCACC |
| *Ov-soxB1* | CAGTCGCAGAAGAACAACCAGGATCGGGTTAAAAGACCTATGAATGCTTTCATGGTCTGGTCCCGTGGACAGAGGCGGAAAATGGCACAGGAAAATCCTAAAATGCACAATTCGGAGATTAGTAAACGACTTGGCGCCGAATGGAAACTATTATCCGAATCTGAGAAGCGACCCTTTATCGACGAAGCTAAACGCCTACGTGCGATCCACATGAAAGAACACCCTGATTACAAATACAGACCCCGGCGAAAGACGAAGACTCTTATGAAAAAAGATAAATACGCGATACCAGGAATGCCACCAGGGGCTCCAGTTCAACAGGTAGGCCGAGAGATGTACCAGATGAACGGGTACATGCCGAACGGCTATCCTATGATGCCCCCTGATCCGAACGCTTACCATCAACACATGTCGAACCCAATGTTGGGCGGACAATATGGATATAACATTCCAACTCAACCCATGTCAACACAGATGACCACCGGTTCATATATGAACGGCAGCTCCAGCTACACCATGACAATGGCCCCATACTCAATGTCCCCATCTCAGGTGCCTCAAAT |
| *Ov-syt* | GCAGGAGAGGGAAGAAAGATGGCAAAAAGGGGCTAAAAGGAGCAGTTGACCTCAGAAGTGTGCAGATTCTGGGCAATTCCATCAAAGAAAAGCCGGACTTAGAGGAGCTGCAAATGAATATGGAAGAGAATGAAGATGCTGAAAGTACAAAATCTGAAGTAAAATTAGGGAAACTCCAATATTCAATGGACTACGATTTTCAGAAAGGCGAGCTAACAGTAAATGTAATACAGGCTGCCGACCTACCTGGGATGGATGTGTCTGGGACGTCTGACCCATACGTCAAAGTCTATCTAATGCCCGACAAAAAGAAGAAATTTGAAACAAAAGTCCATCGGAAAACACTGAATCCAGTATTTAATGAATCCTTCACATTTAAGAACGTACCTTATGCTGATATTACGGGTAAGACGCTAATATTTGCGATCTACGATTTTGATCGCTTTTCAAAGCATGACCAAATTGGTCAAGTCCAAGTTCCAATGAATTCTATAGATCTTGGATCAGTAATTGAAGAATGGAGGGATCTCACCAGTCCTGATAATGATGCTGAGAAAGAAAACAAGCTTGGTGATATATGTTTCTCATTGCGCTATGTCCCCACTGCTGGCAAATTAACTGTGGTCATCCTGGAAGCCAAAAACCTCAAGAAGATGGATGTAGGAGGATTATCAGATCCTTATGTTAAGATATCTCTGATGCTAAACGGTAAAAGAATTAAGAAGAAGAAAACCACTGTCAAGAAATGTACACTGAATCCATACTACAATGAATCATTTGCGTTTGAAGTCCCCTTTGAACAAATACAGAAAGTGTCCCTTTACATCACAGTCGTTGACTACGATCGTATCGGGACGTCCGAACCTATCGGACGAACCTTCCTTGGCTGCAACTCCACTGGTACTGGATTGCGTCATTGGAG |
