# Supplementary material for: Identification of neural progenitor cells and their progeny reveals long distance migration in the developing octopus brain
Source: eLife. 2021 Aug 24;10:e69161. doi: 10.7554/eLife.69161 (PMC8384421; doi:10.7554/eLife.69161)
Supplement: Supplementary file 5. [file elife-69161-supp5.docx]

# Supplementary file 5

**Table S5. Accession numbers of protein sequences used for phylogenetic tree construction**

| **NAME** | **NCBI accession number** | **NAME** | **NCBI accession number** |
| --- | --- | --- | --- |
| *Ac-ASCL1* | XP_005110606.1 | *Lg-ASCb* | XP_009064339.1 |
| *Ac-ELAV-like1* | XP_005092587.2 | *Lg-ELAV* | XP_009047849.1 |
| *Ac-ELAV-like2* | AY956294.1 | *Lg-NEURO-bHLH* | XP_009047744.1 |
| *Ac-NEURO-bHLH* | XP_005089777.1 | *Lg-SOX* | XP_009044439.1 |
| *Ac-POLY-A-BINDING4-like* | XP_005113260.1 | *Mm-ELAV-like1* | NP_034615.2 |
| *Ac-SOX2-like* | XP_005093681.1 | *Mm-ELAV-like2* | EDL30947.1 |
| *Am-ASH-T3* | XP_026298936.1 | *Mm-ELAV-like3* | AAH52097.1 |
| *Am-ELAV-like2* | XP_006571248.1 | *Mm-ELAV-like4* | AAH52451.2 |
| *Am-SOX2* | XP_026300481.1 | *Mm-MASH1* | NP_032579.2 |
| *Am-TAP* | XP_001120974.1 | *Mm-MASH2* | NP_032580.2 |
| *Bl-ASCL1a1* | BL13732 (Ensemblmetazoa) | *Mm-MASH3* | NP_064435.1 |
| *Bl-ELAV* | ASW25830.1 | *Mm-MASH4* | NP_001157086.1 |
| *Bl-NEUROD1-like* | BL07636 (Ensemblmetazoa) | *Mm-MASH5* | NP_001257538.1 |
| *Bl-SOXB1a* | ALH22041.1 | *Mm-NEUROD1* | NP_035024.1 |
| *Bl-SOXB1c* | ASW25831.1 | *Mm-NEUROD2* | NP_035025.3 |
| *Ce-EGL-13* | NP_001294841.1 | *Mm-NGN* | AAC52856.1 |
| *Ce-ELAV* | NP_496057.1 | *Mm-POLY-A-BINDING3* | NP_001157308.1 |
| *Ce-HLH14* | NP_495131.3 | *Mm-SOX1* | NP_033259.2 |
| *Ce-HLH6* | CAA87416.1 | *Mm-SOX14* | NP_035570.1 |
| *Ce-NEUROD1* | NP_498115.1 | *Mm-SOX2* | NP_035573.3 |
| *Ce-SOX2* | NP_741836.1 | *Mm-SOX21* | NP_808421.1 |
| *Cg-ASCL1a* | XP_011413561.1 | *Mm-SOX3* | NP_033263.2 |
| *Cg-ELAV-like1* | XP_011437135.1 | *Mm-SOX4* | NP_033264.2 |
| *Cg-NEUROD6A* | XP_011439492.1 | *Mm-SOX6* | AAH67407.1 |
| *Cg-SOX2* | XP_011455662.1 | *Mm-SOX7* | NP_035576.1 |
| *Ct-ASH1* | ACS36111.1 | *Mm-SOX8* | NP_035577.1 |
| *Ct-ASH3* | ELT88014.1 | *Mm-TCF1* | NP_033357.1 |
| *Ct-ELAV1* | ELU12456.1 | *Nv-ASH2* | XP_032226875.1 |
| *Ct-ELAV2* | FJ830867.1 | *Nv-ASH3* | XP_032237760.1 |
| *Ct-NEUROD* | AST23028.1 | *Nv-ELAV* | LT795588.1 |
| *Ct-SOXB* | AST23030.1 | *Nv-SOX9* | XP_001630037.1 |
| *Dma-ASCL* | AEH21241.1 | *Nv-SOXB1* | SJX71971.1 |
| *Dma-ASE* | AEH21242.1 | *Nv-SOXB2* | ABA02364.1 |
| *Dma-ASH-T3* | XP_032780864.1 | *Nv-SOXF1* | ABA02366.1 |
| *Dm-AC* | NP_476824.1 | *Ob-ASCL1* | XP_014781608.1 |
| *Dma-ELAV2* | KZS07880.1 | *Ob-ASH4* | KOF70326.1 |
| *Dm-ASE* | NP_476694.1 | *Ob-ELAV-like1* | XP_014788038.1 |
| *Dma-SOX1a-like* | XP_032796962.1 | *Ob-ELAV-like4* | XP_014767548.1 |
| *Dma-TAP* | XP_032792341.1 | *Ob-NEUROD1-like* | XP_014775193.1 |
| *Dm-ELAV* | P16914.1 | *Ob-NGN* | HM369392.1 |
| *Dm-LSC* | NP_476623.1 | *Ob-POLY-A-BINDING-like1* | XP_014773726.1 |
| *Dm-PAN* | AAC47464.1 | *Ob-SOX14-like* | XP_014789971.1 |
| *Dm-POLY-A-BINDING* | NP_476667.1 | *Ob-SOX2-like* | XP_014780771.1 |
| *Dm-SC* | NP_476803.1 | *Ob-SOX5-like* | XP_014770685.1 |
| *Dm-SOX100B* | NP_651839.1 | *Ob-SOX9* | XP_014772207.1 |
| *Dm-SOX102F* | NP_726612.1 | *Ov-TCF1-like2* | XP_014780407.1 |
| *Dm-SOX14* | NP_476894.1 | *Pd-ASH1* | CAQ57532.1 |
| *Dm-SOX15* | NP_523739.2 | *Pd-ELAV* | ABO93208.1 |
| *Dm-SOX21a* | NP_648694.1 | *Pd-NEUROD* | CAQ57533.1 |
| *Dm-SOX21b* | NP_648695.1 | *Pd-NGN* | FM163172.1 |
| *Dm-SOXN* | CAB64386.1 | *Pd-SOXB* | CAY12631.1 |
| *Dm-TAT* | NP_524124.1 | *Pd-SOXC* | CAY12635.1 |
| *Dr-ELAV-like1* | XP_017213144.1 | *Pd-TCF/LEF* | ANS60442.1 |
| *Dr-ELAV-like2* | NP_001002172.2 | *So-ELAV1* | HE956712.1 |
| *Dr-ELAV-like3* | NP_571524.1 | *So-ELAV2* | HE956713.1 |
| *Dr-ELAV-like4* | AAH65965.2 | *So-SOXB1* | AGL08098.1 |
| *Dr-NEUROD1* | NP_571053.1 | *So-SOXB2* | AGL08097.1 |
| *Dr-NGN1* | NP_571116.1 | *Sp-ASH1* | XP_003729788.1 |
| *Dr-POLY-A-BINDING1a* | AAH99992.1 | *Sp-ASH5* | XP_003726868.1 |
| *Dr-SOX2* | NP_998283.1 | *Sp-ELAV-like3* | XP_011675251.1 |
| *Dr-ZACH1a* | NP_571294.1 | *Sp-NEUROD4* | XP_030846597.1 |
| *Dr-ZACH1b* | NP_571306.1 | *Spo-ASCa* | KJ493812.1 |
| *Es-ASCL* | cluster_17772 (Belcaid 2019) | *Spo-ASCb* | AIZ72740.1 |
| *Es-ELAV-like2/4* | cluster_2695 (Belcaid 2019) | *Spo-ELAV1* | AIZ76501.1 |
| *Es-NEUROD1-like* | Cluster_3866 (Belcaid 2019) | *Spo-NEUROD* | KJ493814.1 |
| *Es-SOX2-like* | cluster_17675 (Belcaid 2019) | *Spo-SOXB1-2* | KJ493809.1 |
| *Hd-ASCL1* | OQV19415.1 | *Sp-SOXB1* | NP_999639.1 |
| *Hd-ELAV-like2* | OQV23475.1 | *Tc-ASH1* | NP_001034537.1 |
| *Hd-NEUROD1* | OWA52745.1 | *Tc-ELAV-like3/4* | XP_015833241.1 |
| *Hd-SOX2* | OQV14806.1 | *Tc-SOX3* | XP_008193147.1 |
| *Lg-ASCa3* | XP_009065285.1 | *Tc-TAP* | XP_970244.1 |
