# Supplementary material for: Identification of neural progenitor cells and their progeny reveals long distance migration in the developing octopus brain
Source: eLife. 2021 Aug 24;10:e69161. doi: 10.7554/eLife.69161 (PMC8384421; doi:10.7554/eLife.69161)
Supplement: Supplementary file 6. [file elife-69161-supp6.docx]

# Supplementary file 6

**Table S6. Protein sequences of *O. vulgaris* homologs used in phylogenetic tree reconstruction**

| *Ov*-ELAV | MDSQIIQQNGGNHSPISTGTQMDDRDNVSKTNLIVNYLPQTMTQEEIRSLFASIGEVESCKLIRDKPTAMNTLRQNDLDQGLPETANTDFHCDLLPAARNASTDAEKCQSLGYGFVNYKYPSDAEKAINTLNGLRLQNKTIKVSYARPSSESIKGANLYISGLPKSFTQLDLEKLFSQCGFIITSRILYDNNTGLSKGVGFIRFDQRIEAERAIQKLHGTIPEGATEPITVKFANSPSSNKNAVPLALAASYLSPSRRIFGPIHHAAGRFRYSPLEASLLPGTILPGNALNGTGWCIFVYNLAPDTEDDVLWRLFGPFGAVQNVKVIRDFQTQKCKGYGFVTMTNYEEALMAIQTLNGFLLGTRVLQVSFKTNSRKS |
| --- | --- |
| *Ov*-ELAV-like1 | MEMDNKRTNLIINYIPQTLTDEEFRSMFLSIGPIKSAKIVRDKATGYSYGFGFVDYENVEDAQRAMQTLNGLQMQNKTIKVALARPGGEEIKGANLYVRNLPRHYQQMDLERLFQQFGTIIQSRVLTDQATGVSKGVGFVLFDQKKQAEEAINQITGTVPAGGTEALLIKFADDNAKKVRPPAMPYINTAPCFPAPGPMRSPGNRFRYNPVTGNYSTPNSGLSLSQGGYVIFVYNIGASSCEKTLWQLFSPFGTVQKVNIMRDHQKGQSKNYGFVTMTNLQEAQNAIARLNGYDYAGCGRPLQVKFKN |
| *Ov*-ELAV-like1B | MATEDHCEDNGGNLTNLIVNYIPQSLSNEEFRAMFLTIGPIKSSKIIQDRATGYSYGFGFVDYEHPEDAARAVQTLNGLHIENKRIKVALARPSGLGVKGANVYVSHLPNHYAQSDLVKLFEPYGIIIRSRIMTHPNTNQSKGVGFVLYDQKKMAEAAIADLNQTIPPGGSEPIIVKFANEAPNSKKTRPPHEQYMNNNNSIQQTFSTPMLMRNERFTSPVPVNYSPNNPNPNPNNDMSNSPVLFIYNTGNMDESDLGQLFCGFGTVQKANIVRVHQKTKGYGFVTMANQHEAENAVGRLNGYRYRGWGPLQVSFKNK |
| *Ov*-PolyA_binding_1-like | MNPTAPNYPMASLYVGDLHPDVTEALLFEKFSSAGPVTSIRVCRDMIARRSLGYAYVNFQQPADAERALDTMNFDAIKGRPIRIMWSQRDPSLRKSGLGNVFIKNLDKSIDNKALYDTFSAFGNILSCKIVSDESGSKGYGFVHFETGEAARCAIEKVNGMLLNGKKVYVGKFIPRRERMQQLGDKYKKFTNVYVKNFNDDLDDDKLRQVFEKFGKIASAKVMKDETGKSKGFGFVSFEEAEAAEAAVNEINGMDMNGRILYAGRAQKKAERQAELKEKFEKIKMERINRYQGVNLYVKNLDDAIDDERLRKEFSQFGTITSARVMTENGRSRGFGFVCFSSPEEATKAVTEMNGRILVFKPLYVALAQQKEERKAQLASQYIQRIQTIRNPLSSINTIFQASNPTSYFVPTMPQQRYYPSPITQLRAPGPRWQSIRPASAQQAAAYPPLATQQMRQTRPTGAQSTVRSNLGSRPITGQTGQRMAVPPSVTGRQTTNMQSQNSRANFKFGPMRPLGPMGTMQSMLGQPTAQQPVVVHGQEPLTSGMLAAAPPQEQKQMLGETLFPLIHGMYPDMAGKITGMLLEIDNSELLHMIDSHESLQAKVEEALAVLQAHQAKEAAATNAGVVKKD |
| *Ov*-NEUROD | MQMFRMPIKSLETEDEMCFGDSSSELLGDGSLLDDTCSEDDLDDYGSEPEDCLEGMTGNEDEQEMSDTKMKKSAKNYPYRRKKEEKPSSKDKNEPRLPKKRGPKKKRMTKARVQKLRLRRVKANARERNRMHGLNDALDTLRLHVPCTSKTQKLSKIETLRLARNYISALGEILKNGTKPDGVSFAKALSKGLSQNTMNLVAGCLQLNPRTLHPDSTLPKTYQYDFSSQIDFGSAVTQIPYPPSSYATTLHHHHHPP |
| *Ov*-NGN | MVMYLNKEVLLGPVSCVDMTGASVKLEPAISDIVSLSVPSSTHSMLTTTTTTIGAITTATTTTTTADFSLSGVLDVVDNVDVKVSSGFSWSNPGALSSVIHSPSSVQSPTLSPESPTTNAFHVDDHLLYNHSQHQNQNIHHRYQPLYPRPEKSMELSTVVDSPLSVSTPSSPSSSSSSSSSSSSSSSSLISSPYSSSSLSFSPLYTGDHKVPALKSNLQQLLPQNQPQNERQPESQLQQESKPQPQQQPDMKAAVACPVLLPTSAAQCKDTVVPTNSINTTAETTTTTASSSSSSSLAAPRTKATTTRRKITEMTKATRPPIISRSPPTSSSSSSSSSSLSSSSPLTSRASPLSSSSPPSSIPIISSSTDSVDGLKRKQVSPEKNCGAKRRNGPTAKKKRYTKSRIRNCNPLIVMKIKRNRRMKANDRERSRMHSLNDALDCLREVLPNYSDEGKLTKIETLRFAHNYIWTLSEALKLMDTAESKGEFGDIPEKLATLGLFARLSQHKHHHHYHHHHHHRQQLQQHPLQLQPQPQQQPLLQRPQPPQQQQVVLQQQSLQLQPKLQPTQPQQPLPHLLPPLVPPPAQQLPLSQLLPTPTPTSPPPPPHQQQQQQVLLANEIEMEEDDEKDVVKSENDDDDDDDDEDEDDDDEDEDDDDDDDGDDDGGDDESDRLLKRVTDTSFLQDFLMSQENEPVTTSGYRQEPNRFRHHHRHHHHHHHHRRLLRREANNYNDGDAVANSNGNSSPLYAPGIYGDYGNKIHAINNNNNNSDMEVTSSSSSSSGGGSRSGCCCSSSSSSSGCGGCSCCISNSSDSGSSSSISSSNSRSRSSSVSSGGSGGSNDNLADSATGVRSSNGTNSPSAPVHDRRPRRNHHHHHHHHHHHPYQLPQHQYHQHQHQPKQQLTKQPQHHHLYFQQQQHQLTVEPEQNESLHFSTDFSTPKSNFDVQDDHFVELTKL |
| *Ov*-ASCL1 | MTAALVPEPMVVESTLTSSATTTTTTATAMMAATTTTNAFSTTAKAFSTTTAVAATNGFPTTTTTAATTNSTTTTTTPNSKSVRTISMKENARELMNCKRRLDFNHTNYLPLHRPQTVAVARRNERERNRVKLINMTFATLREHIPNGPKGSKNKKMSKVETLRAAIDYIHYLQDLVEDSDTVNTIFDSSCLTAAGLTAAGLTNGIYQTTAMNSPRPSSCSDSSYEGLSAEEEELLGFTKWF |
| *Ov*-SOXB1 | METDLSKSSLAPGSGPQSLGQQSNPGANGQGNQGASQSQKNNQDRVKRPMNAFMVWSRGQRRKMAQENPKMHNSEISKRLGAEWKLLSESEKRPFIDEAKRLRAIHMKEHPDYKYRPRRKTKTLMKKDKYAIPGMPPGAPVQQVGREMYQMNGYMPNGYPMMPPDPNAYHQHMSNPMLGGQYGYNIPTQPMSTQMTTGSYMNGSSSYTMTMAPYSMSPSQVPQIKREPNPTGQPSPANRCPTGDLREMISMYLPGDNTPDHTVQSRLQMQTQYAHATTTEGGVNNTVPLTHM |
| *Ov*-SOXB2 | MSKPQAEHVKRPMNAFMVWSRGQRRKMAQENPKMHNSEISKRLGAEWKLLSEEEKRPFIDEAKRLRALHMKEHPDYKYRPRRKPKSLLKKDNKYAFPIPVLPGMNTFGAVPSFSPGSQDSLLASNFGEKARAFLPPTSTTHALYSHLAENAKMDSPLNIRPHELNGASGLYSPYMTSHSSLGSLPPAAHTHSGLTGQYLLPCCPPPYMPSQDLHRPVAYVLVKPEDHYRHTVI |
| *Ov*-SOX11-like | MVPQNTTLVADPTSSSFNQSVMFGSQLVDPGSHTPYTDATNCKKSSSHVKRPMNAFMVWSQIERRKISEVQPDMHNAEISKRLGRQWKLLNEHDRKPFVDEAERLRVLHLQEYPDYKYRPRKKAKLSGKAEGSNNTSNASSVGTTSTSKISKGPGHKTHKTEKNSSNKHRTNGGGGVVKSSNGLSSNGHTSNTANRLKLKLTIDKKFKESIKASKHVSVPSSQLTPPAKVPSSPSLYAPPTPEAVSFYPEEAFETPAASPQELNCVTNATVAGAASAGAFQVISSTQLTATAVDGKLLYQSAQIGGTTTSLPTSTALAALATAGTGAAMVGQQHLSLTGQQTTTDQHQHQHQLQQLQQQQQHNQHMDSVPLADLDSLTDVLQLPNNWHLELGNLDLSKLADAEFNLDMQNQNTPNASHFEFPDYSTPEVTEMIEADWLESGLGTLICKQ |
| *Ov*-SOX5-like | MLSTTSPALSTPVISQSCSSSKISSPISVESRRVSSSPHIATSPYHLTAPSIPRTLPSPSLQHHSPVSSPHANPAMATTATAVQANNIWANNSTGYNSSVAVAAAAAVAAAAAAAASDQDMPLNLSKPRYEMKLEKSSSEDSVNLSASQSKVEAIITPPPAHSNHQQRLPLTSSPTEMSALVGSRTPFGVAPPPQYVSSPYLGLPNHAAVPVGLSLNNLSPHTTSLNGKPSPSESEKDFFYSQDSLVQEVLVRQLSQNVNNQVFPMHLPMYAVPSTTMPQLSQLAACKDNRVMMPAEMSHEENHTYVQHLQSKMFGAKIIRAHKEKPDPSKPHVKRPMNAFMVWAREERRKILKACPDMHNSNISKILGAKWKAMSNAEKQPYYEEQSRLSKLHMEKHPDYRYRPRPKRTCIVDGKKLRISEYKALMKSRRQDIRRVWYNESGTTYVEGSENSSPYDTNQLLGSNMSGKDGKDSEFQGKLKSDETNSEDGDGSDINDIMSDVSLDASYSTAGSNSSHMQLGEVAASVS |
| *Ov*-SOX9-like | MSDTEESRGSPGEHSSPDTCGMEDSMSGSGLFSADHRFPQQIQDAVSQVLKGYDWTLVSMPSRTNGGEKRKPHIKRPMNAFMVWAQAARRKLADQYPHLHNAELSKTLGKLWRLLNEKEKRPFIDEAERLRVQHKKDYPDYKYQPRRRKPLKTSGVSNSGSVDVQHHLPPGMMLKSLQNSPTPHMSDGESSNCSSPHNGSHGPPTPPTTPNQQELIKCMADRSRLRSMGGLHSQAHPIDFSRVDLRELGNEVMSLDNIDDHELDQYLAMNPGAILSHQHNVAAAAAAATAAAAAQSDNGYTCYNQVSPTAVSTAPTWTSNYRVSASACVQPFNNGSPTSIPYDLSSQQQQQQQQCF |
| *Ov*-TCF1-like2 | MPHVSSGGSEDFFSKDEVKVYKDEGEEEKRSSENLSEDKLGLVTESEEGKNSSLPGQSYPGGEKSNTSGRPEDDKVVSQHPADRGIGPIPGYVVSPYPYPNGASVPASLGGKMSMVQPPLASLMMYNNDYSQPPPAHMGIPPVHIDPKTGLPRPPMYGYPAPPGQFPHPIYGPDFPQVQWQRPPGYPISSGAFSGPYPPSLINTSPLTRFGPPSLLPPPGLPHPGIPHPAIVTAGPKQEVMSHSQDNHRHSLQEQSSSHASEAQTAGDKKKPHIKKPLNAFMLFMKEMRPKVVAECTLKESAAINQILGRKWHALDRAEQAKYYEMARKEKELHMQLYPGWSARDNYASHTKKKKRKRETTGEDEAKARLPWYFNDVTTDSITAEGSSGGDSYQWERTRPSIIECSNPKKCRARFGLDQQNQWCKPCRSMHEKCDQTADLETKISTEV |
